# Optimal Resource Allocation to Survival and Reproduction in Parasitic Wasps Foraging in Fragmented Habitats

**DOI:** 10.1371/journal.pone.0038227

**Published:** 2012-06-06

**Authors:** Eric Wajnberg, Patrick Coquillard, Louise E. M. Vet, Thomas Hoffmeister

**Affiliations:** 1 INRA, Sophia Antipolis Cedex, France; 2 Netherlands Institute of Ecology (NIOO-KNAW), Wageningen, The Netherlands; 3 Institute of Ecology, FB 2, Biology, University of Bremen, Bremen, Germany; California State University Fullerton, United States of America

## Abstract

Expansion and intensification of human land use represents the major cause of habitat fragmentation. Such fragmentation can have dramatic consequences on species richness and trophic interactions within food webs. Although the associated ecological consequences have been studied by several authors, the evolutionary effects on interacting species have received little research attention. Using a genetic algorithm, we quantified how habitat fragmentation and environmental variability affect the optimal reproductive strategies of parasitic wasps foraging for hosts. As observed in real animal species, the model is based on the existence of a negative trade-off between survival and reproduction resulting from competitive allocation of resources to either somatic maintenance or egg production. We also asked to what degree plasticity along this trade-off would be optimal, when plasticity is costly. We found that habitat fragmentation can indeed have strong effects on the reproductive strategies adopted by parasitoids. With increasing habitat fragmentation animals should invest in greater longevity with lower fecundity; yet, especially in unpredictable environments, some level of phenotypic plasticity should be selected for. Other consequences in terms of learning ability of foraging animals were also observed. The evolutionary consequences of these results are discussed.

## Introduction

Thanks to modern agricultural methods, urbanization and climatic change, natural ecosystems are increasingly suffering from fragmentation leading to both modifications in community structure and function, and to a loss in biodiversity due to species extinction [1]–[7]. Insect species, and especially parasitic wasps, can be especially adversely affected because of their typical small size and low population densities [8]–[9], and also because they usually lag behind their hosts in discovering isolated habitat fragments [10]–[11].

Besides potentially impacting parasitisation success, habitat fragmentation can have important evolutionary consequences for host-parasitoid interactions. This is because interacting species are essentially dynamic entities with both inter-individual genetic variation and intra-individual phenotypic plasticity responses to environmental change [3]. For example, increased fragmentation might act on the way resources are allocated to either fecundity or longevity in parasitoid females [12], likely resulting in disruptive selection for individuals with either low fecundity and increased lifespan and dispersal ability, or a high fecundity with a low survival potential [3]. Life expectancy and the number of eggs available to be laid, henceforth termed egg load, are indeed the main components of parasitoid fitness and thus subject to strong selective constraints [13]–[18]. In turn, such selective constraints can lead to evolutionary changes in reproductive decisions, impacting both population dynamics and stability of host-parasitoid interactions [19]–[20], and thus, *e.g.*, the outcome of biological control programs [21].

Particularly in synovigenic species, in which females have the ability to mature eggs throughout their life, a dynamic control of egg load can enable animals to retain some flexibility during the adult stage to minimize their risk of experiencing time- or egg-limitation [16], [22]–[24]. In some species, egg load can be dynamically adjusted to environmental conditions by means of host feeding that provides nutrients to mature more eggs (and also to live longer; [25]–[27]), and/or by egg resorption, recycling unlaid eggs to retrieve valuable nutrients [14], [16], [23].

From an evolutionary point of view, life expectancy and egg production are constrained by a trade-off between survival and reproduction [24], [28]–[29] and a significant number of both theoretical and experimental studies have tried to identify the factors involved [14], [29]. One of the main arguments is that parasitoid wasps, even if they feed on hosts, have a limited amount of reserves from which they draw to produce eggs at the expense of other functions such as somatic maintenance, and thus survival [12], [30]–[32]. Reproduction and survival thus compete for the same resources leading parasitoids to dynamically trade current for future reproduction [17], [29].

Trade-offs play a central role in evolutionary biology [32], shaping the way animals can optimally allocate their resources in different habitats [22], [24], [31]. Among them, the trade-off between survival and reproduction has arguably been the most studied [17], [29], [30], [32], [33]. Accurately unravelling the selective constraints foraging animals face leads to understand how allocation of resources to either survival or reproduction can vary during the life of foraging females [25]. Moreover, since animals cannot have a perfect prior knowledge of their habitat, such phenotypic plasticity is usually associated with some learning ability through which parasitoid females can adjust their behaviour according to the information they acquire from their environment [17], [34], [35], especially their rate of host encounter [17], [36].

In fragmented habitats, animals will have to cover longer travel distances between patches of resources and will thus most likely be selected for higher dispersal ability than in continuous habitats. In turn, this will most likely lead animals to be selected for longer survival and lower fecundity. Reciprocally, in landscapes with less isolated resource patches, travel distances between patches of resources will be reduced and animals will be expected to invest in higher fecundity rather than survival rate. Some of these predictions were verified experimentally by [12] on *Asobara tabida*, a solitary parasitoid of *Drosophila* larvae. Wasps living in habitats where patches of hosts were widely spaced indeed had lower egg loads and higher fat reserves, providing them with more energy to be spent on travel and survival, than individuals living in habitats where resources were more accessible. Plasticity for energy allocation was present in populations from both kinds of habitats [12].

Provided that there is phenotypic plasticity along a trade-off between survival and reproduction in parasitoid females, which is linked to information processing of habitat quality, the central question will be how plastic the allocation of energy to reproduction or survival should be, given that such plasticity in a life-history trade-off is costly [37]. It seems obvious that variability of host availability and isolation of habitat patches should select for such plasticity. However, whether the benefits of such plasticity can outweigh the possible associated costs and the reallocation of limited resources along the life-history trade-off has seldom been addressed. Hence, what should the optimal life history strategy be for parasitic wasps foraging for hosts in habitats showing different levels of fragmentation? More specifically, what are the evolutionary consequences of habitat fragmentation on the reproductive strategies of parasitic wasps? We predict that habitat fragmentation (1) should lead foraging parasitoid females to invest in higher survival rate with lower fecundity, and (2) should influence their level of phenotypic plasticity.

In order to address these questions, a Monte Carlo model was developed to simulate the exploitation of habitats with different levels of fragmentation by synovigenic parasitoid females laying one egg per host, without taking into account host-feeding, sexual reproduction or potential sources of larval or adult mortality. Optimal reproductive strategies were identified by means of a genetic algorithm [38]–[39]. The results demonstrated that the level of habitat fragmentation has a strong influence on the reproductive decisions parasitoid females should adopt along a survival-reproduction trade-off. For instance, in habitats that are more and more fragmented, parasitoid females will indeed most likely be selected to have greater longevity and lower fecundity. Optimal levels of phenotypic plasticity and learning ability also appeared to be influenced by the level of habitat fragmentation. The evolutionary repercussions of these results are discussed.

## Description of the Model

The word “patch” usually defines a spatial subunit of the foraging area in which resources are aggregated [40]–[41]. In the case of insect parasitoids, host patches may range from single rotten fruits or mushrooms in which potential hosts are developing, or host egg masses for egg parasitoids to leaves or entire plants. In the present study, however, in which we address the effect of habitat fragmentation, we used the term “habitat patch” to define a spatial subunit of a patchy fragmented landscape that may contain hosts and is surrounded by landscape matrix devoid of hosts. The concept of “patch” and “habitat patch” both describe the distribution of resources in the foragers’ habitats in a similar fashion, but at different spatial scales. We decided to use the latter in order to explicitly phrase the question addressed in this work in terms of habitat fragmentation. More precisely, in the present study, environments with habitat patches of low quality (*i.e.*, containing a lower number of resource items) and separated by large distances (*i.e.*, implying longer time to reach them) correspond to isolation of habitat patches in a fragmented habitat.

To find optimal reproductive decisions for the life-history trade-off between reproduction and somatic maintenance and the level of phenotypic plasticity in the allocation along the trade-off, we developed an individual-based, non-spatially explicit model simulating, over several generations, the reproductive trajectory of individual parasitoid females exploiting hosts distributed in habitat patches in their environment. The model is discrete in time and integrates both stochastic and deterministic components. During their life, simulated wasps encounter habitat patches containing a number of hosts depending on the feature of the environment they are foraging in. Different habitat patch qualities were compared: 25, 50, 100, 250, 500, 750 or 1000 hosts.

The number of habitat patches encountered during one generation by a simulated female depends on the time she spends travelling between them, on the time she allocates to each of them, and on her total lifetime duration. Different inter-patch travel times were compared: 100, 200, 300, 400 or 500 time steps. Once in a patch, the foraging female attacks hosts and produces an increasing number of progeny, with a rate of progeny production decreasing with time to take into account patch depletion. The cumulative number of progeny produced on a patch at time *t*, *N_t_*, was computed using the following equation:
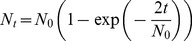
(1)where *N*_0_ is the initial number of hosts in the patch. Similar exponential models are regularly used as a fitness function in the literature (*e.g.*, [42]–[44]).

Simulated females left each habitat patch they exploit at the optimal time predicted by the marginal value theorem (MVT; [45]), *i.e.*, when their local rate of progeny production fell below the estimated rate in the environment as a whole. Such an optimal patch-leaving policy was adopted since the goal of the model was to look for optimal reproductive strategies, and because most foraging animals, especially insect parasitoids, usually behave in qualitative agreement with the marginal value theorem [41], [46]–[47]. Actually, in the model, animals leave a habitat patch if they have a sufficient remaining lifespan to reach the next patch. If this is not the case, they remain (*i.e.*, longer than what is predicted by the MVT). Such a patch-leaving rule, which is re-evaluated at each time step as soon as a female reaches the optimal time to remain in a habitat patch, approaches the optimal prediction of a dynamic version of the MVT [48].

Animals forage in environments that might have different levels of stability across generations, where stability describes the continuous ability of a foraging animal to encounter resources in the habitat it is born into. We thus added into the simulation model a parameter *p* describing the stability of the environment, and which corresponds to the probability of a female to start her life in a habitat patch containing hosts. A female born in a habitat patch devoid of hosts thus has to start her life by dispersing and has thus to invest into a travel time before reaching the first habitat patch to exploit. Different values of *p*, corresponding to different levels of environmental stochasticity, were compared: 0.00, 0.25, 0.50, 0.75 or 1.00.

**Figure 1:**
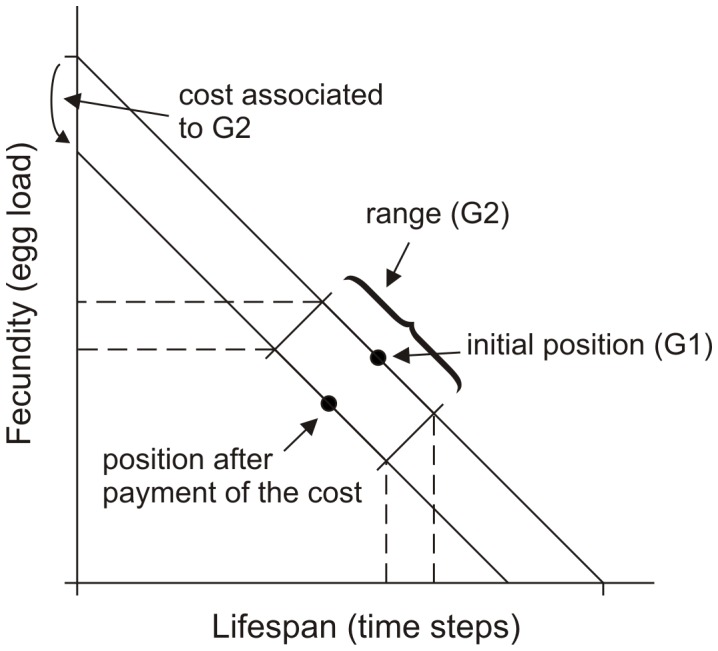
Trade-off between lifespan and egg load describing the main parameters used in the simulation model. The initial reproductive strategy is defined by G1 and each animal has a certain phenotypic plasticity defined by the range G2, but pays a linearly proportional cost for it, both in survival time and egg load. A third parameter G3 (not shown) defines a learning ability used by the animal to move along such a phenotypic plasticity. Optimal values of the parameters G1, G2 and G3 in different habitats are estimated by means of a genetic algorithm (see text).

Animals in the model are assumed to follow a linear trade-off between lifespan and egg load as has been experimentally observed by [31] and [49], and used in theoretical studies by several authors (*e.g.*, [22], [28], [50]). Hence, the model assumes that animals have a limited amount of resources that are allocated either to somatic maintenance and survival or to the production of eggs. The range of such a trade-off was arbitrarily defined to be between 0 and 1000 time steps for longevity, and between 0 and 1000 eggs for egg load. Using different values would lead to a change in scale without qualitatively affecting the results obtained. The reproductive strategy used by each animal along the trade-off was described by a parameter G1 (see Fig. 1), which we expressed in terms of longevity, defining the end point of each simulated generation, the animal running out of either time or eggs to lay.

In the model, like in real situations ([13], [26], [51]), the reproductive strategy adopted by animals has been considered to be dynamic, enabling females to accurately adjust their survival time and egg load along the trade-off between longevity and fecundity. For this, phenotypic plasticity has been added through a parameter G2, expressed in units along the trade-off, defining a range of possible strategies (see Fig. 1). Hence, low values of G2 simulate rather proovigenic parasitoids that have matured the majority of their eggs at emergence and that cannot reallocate the energy to increase fecundity or lifetime [52], [53]. At the other extreme, higher values of G2 represent synovigenic parasitoids that mature eggs throughout their life and are able to instantaneously reallocate energy to either fecundity or lifetime depending on host availability [54].Phenotypic plasticity can entail a cost in animals [37], [55], which has rarely been taken into account. Such a cost can be due, *e.g.*, to the females requiring to maintain additional sensory and information processing machinery and/or conducting the necessary physiological adjustments [56]–[57]. Hence, in the model, animals having phenotypic plasticity paid a linearly proportional cost, *c*, both in survival and egg load (see Fig. 1). Without such a cost, all animals will have the highest possible phenotypic plasticity along the longevity-fecundity trade-off. Different costs were compared: 0.1, 0.2, 0.3, 0.4 or 0.5, expressed as the proportional effect of the phenotypic range compared to the maximal possible phenotypic plasticity and translated in terms of survival and egg load (see Fig. 1). For example, a cost of 0.5 with a maximal possible phenotypic plasticity will constrain females to half of both their maximal possible lifetime and egg load. Since the cost was supposed to act mainly on the need to maintain the plastic machinery, we assumed that it remained constant across environmental situations [55]. Also, such a cost of plasticity could have been taken into account in a non-symmetrical way, having more effect on one fitness component than on the other [24]. Following [29] and [58], we decided to consider a cost acting symmetrically on longevity and egg load, so that effects on both traits kept the same magnitude and could be compared directly.

Within the phenotypic plasticity range defined by G2, simulated females choose the most adapted reproductive strategy according to a learning ability based on their past host encounter rates. For this, as in many other studies (see [59] for a review), we used an updating process based on a linear operator [60]. Animals start their life with a prior estimate of their host encounter rate, *μ*_0_, that was fixed to the midpoint between their lower (*i.e.*, 0.0, when they are travelling between patches) and higher (*i.e.*, 1/*t*_1_, where *t*_1_ is the time to find the first host on a newly discovered habitat patch) possible instantaneous host encounter rates. Then, at each time step throughout their life, they compute a new instantaneous host encounter rate, *λ_i_*, and update their overall estimate of host encounter rate, *µ_i_*, using the following equation:
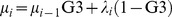
(2)where G3, satisfying 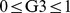, is called the memory factor and gives the weight of the past. Within the range of phenotypic plasticity, the higher the estimated host encounter rate, the more animals will invest in egg load with a lower longevity. When the reproductive strategy of animals is optimized (see below) such a learning mechanism coupled with a phenotypic plasticity will lead parasitoid females to die at the exact moment they lay their last egg. Indeed, if females still have eggs to lay but do not encounter hosts, their learning ability will lead them to convert these eggs into survival in order to find additional hosts in the future. Reciprocally, if females still have some remaining time to live but no more eggs, they will trade dynamically longevity for new eggs to lay. Table 1 lists all parameters of the model, with the values used. The success provided by each reproductive strategy was quantified during 20 successive generations of a parasitic wasp. The global fitness of different strategies is usually quantified by the geometric average of reproductive output over several generations [61]. In the present study, we used the arithmetic average number of progeny produced per generation for the simulated females to quantify their global reproductive success. Preliminary trials showed that results were the same as those obtained when a geometric mean is used.

**Table 1:** Definition of the model parameters with the values used.

Symbol	Values used	Meaning
*N*_0_	{25, 50, 100, 250, 500, 750, 1000}	Initial number of hosts per habitat patch
*t*		Current time on patch (discrete)
*N*_t_		Cumulative number of progeny produced at time *t* by a female on a habitat patch
*T*	{100, 200, 300, 400, 500}	Time steps spent travelling between habitat patches
*p*	{0.00, 0.25, 0.50, 0.75, 1.00}	Probability of a female to born on a habitat patch
*c*	{0.1, 0.2, 0.3, 0.4, 0.5}	Cost associated to the phenotypic plasticity
*μ_0_*		Initial prior estimate of host encounter rate
*µ_i_*		Updated estimate of the overall host encounter rate
*λ_i_*		Current instantaneous estimate of host encounter rate
G1		Initial position of the female on the trade-off between longevity and fecundity (expressed in terms of longevity)
G2		Range of phenotypic plasticity (expressed in units along the trade-off between longevity and fecundity)
G3		Memory factor in the learning process giving the weight of the past

Our model was not based on the biology of a particular parasitoid species but can be applied to any generalist or specialist species exploiting hosts distributed in depletable patches in the environment. More precisely, our model simulates the reproductive behaviour of thelytokous (*i.e.*, without sexual reproduction), solitary (*i.e.*, laying only one egg per host) and synovigenic females that are not suffering from larval mortality and without superparasitism (*i.e.*, not re-attacking already parasitized hosts). Compared to real animals, however, our model makes four main simplifications. Firstly, we assume that animals have an instantaneous vitellogenic activity enabling them to mature eggs instantaneously [23], although, in some parasitic wasp species, egg maturation rate can be very low [25] and is sometimes even considered to be one of the main constraints acting on synovigenic species [24], [26]. However, generally insects are noteworthy, relative to many other animals, for the speed with which they can mature eggs readied to be laid [23], [62], justifying, at least qualitatively, the way the egg maturation process was included in our model. Secondly, simulated females in our model do not feed on their hosts to replenish their resources, as this is actually the case for several parasitoid species. Other theoretical studies also assume non-host-feeding parasitic wasps (*e.g.*, [22]). Thirdly, the parasitoid females have a constant host searching ability throughout their life, although it was shown that foraging behaviour can sometimes depend on egg load [63]. Finally, simulated females did not experience a mortality risk while travelling between patches of hosts in their environment [5]. All simplifications were adopted to keep the model as simple and neutral as possible, without too much loss of generality. As a result, the model enables to identify, in each tested situation, the reproductive strategies leading individuals to maximize their reproductive output.

In each environmental situation tested, values of the three parameters G1, G2 and G3 that maximize the reproductive success of the simulated animals were identified by means of a genetic algorithm [64]. Although remaining computationally simple, such a numerical method, that has been used to solve several other ecological questions (*e.g.*, [39], [65]–[66]), allows us to find in a flexible way optimal or close to optimal solutions to problems, even difficult ones [38], [67]–[69]. Several types of genetic algorithm are available. The one we used is similar to the GENITOR algorithm that has been demonstrated to be highly efficient in optimizing stochastic processes [70]. A population of 100 chromosomes was defined, each coding for three genes corresponding to the three parameters G1, G2 and G3 whose values are used to evaluate the fitness of the simulated females. Chromosomes leading to lowest fitness are replaced by the offspring of the fittest ones. In the process, chromosomes are randomly modified through both recombination and mutation enabling the exploration of new solutions. Repeating these steps, genetic algorithms reach optimal solutions [64], [71]. In our case, a mutation rate of 2.5% per gene and a recombination rate of 60% were used, and the process was repeated for 500 cycles. Some pilot studies indicated us that such recombination and mutation rates were those leading rapidly to an evolutionary stable solution, and indeed 500 cycles were enough to reach such a solution for all the situations we compared. In order to avoid reaching local optima, each situation was optimized 10 times. In each case, the solution leading to the highest reproductive success was considered to be the optimal solution and was used in the results [69]. All combinations of all possible values of (1) habitat patch quality, (2) travel time between habitat patches, (3) probability of the female to be born in a host-containing habitat and (4) cost associated to phenotypic plasticity were compared, representing a design consisting of 7×5×5×5 = 875 situations. The effect of each of these factors on optimized values of the three parameters G1, G2 and G3 was analyzed with 4-ways ANOVA with all possible interactions involving at most two factors. Such statistical analysis produced a large number of significant effects. We refrain from discussing all of them, but focus on the most statistically significant results.

## Results

**Figure 2:**
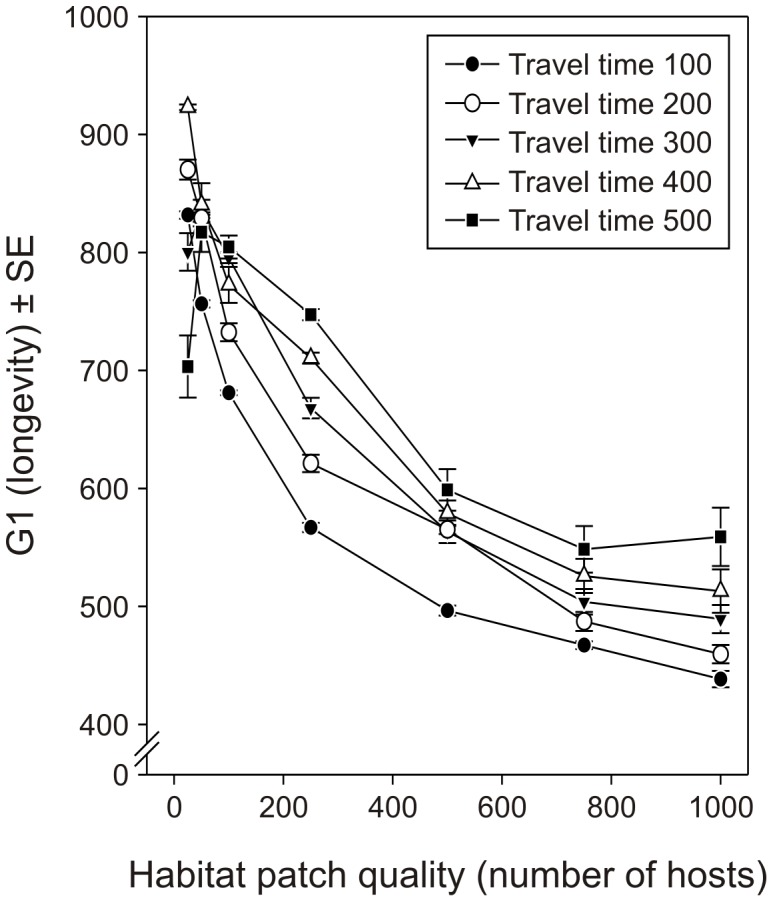
Effect of habitat patch quality and inter-patch travel time on the optimized values of the parameter G1. Average (±SE) optimized values for the parameter G1 defining the initial reproductive strategy on the trade-off between longevity and egg load (see Figure 1) for parasitoid females foraging for hosts in environments with different habitat patch qualities and different inter-patch travel times.

**Figure 3:**
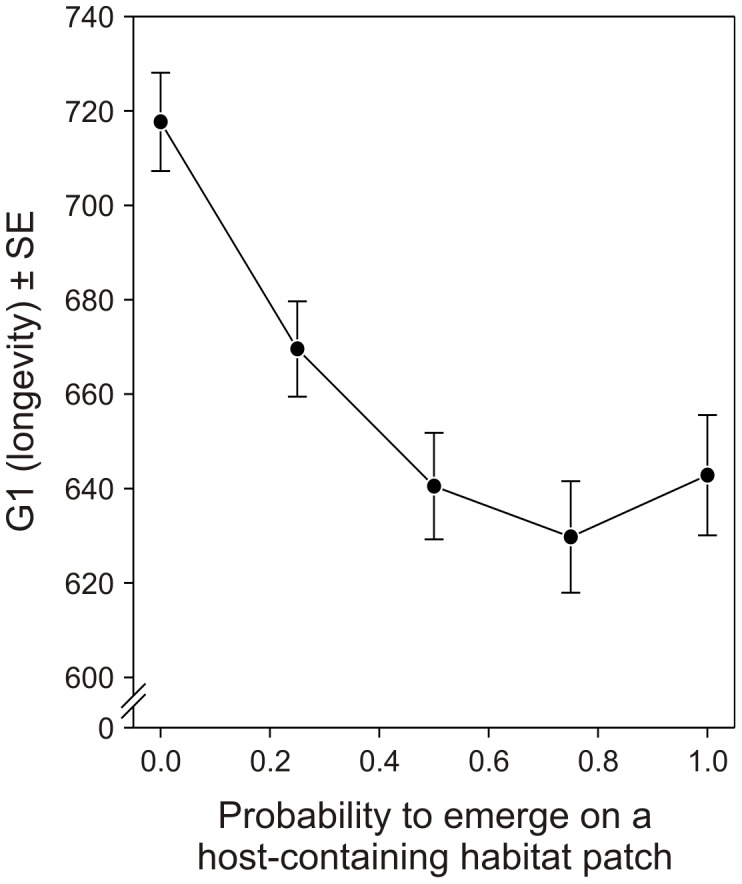
Effect of the probability for a parasitoid female to emerge on a host-containing habitat patch on the optimized values of the parameter G1. Average (±SE) optimized values for the parameter G1 defining the initial reproductive strategy on the trade-off between longevity and egg load (see Figure 1) for different probabilities for the parasitoid females to start their life on a habitat patch.

Parasitoid females should invest less in fecundity and more in longevity when they are foraging in habitats that are more fragmented, *i.e.*, with smaller habitat patches and with longer travel times to reach them (effect of habitat patch quality: F_6,736_ = 1292.72, *p*<0.0001; effect of travel time: F_4,736_ = 106.37, *p*<0.0001; Fig. 2). Furthermore, the higher the probability that a female starts her life in a host-containing environment, *i.e.*, the lower the probability of initial dispersing to a new habitat, the more the parasitoid should invest in higher fecundity with lower longevity (F_4,736_ = 111.01, *p*<0.0001; Fig. 3).

**Figure 4:**
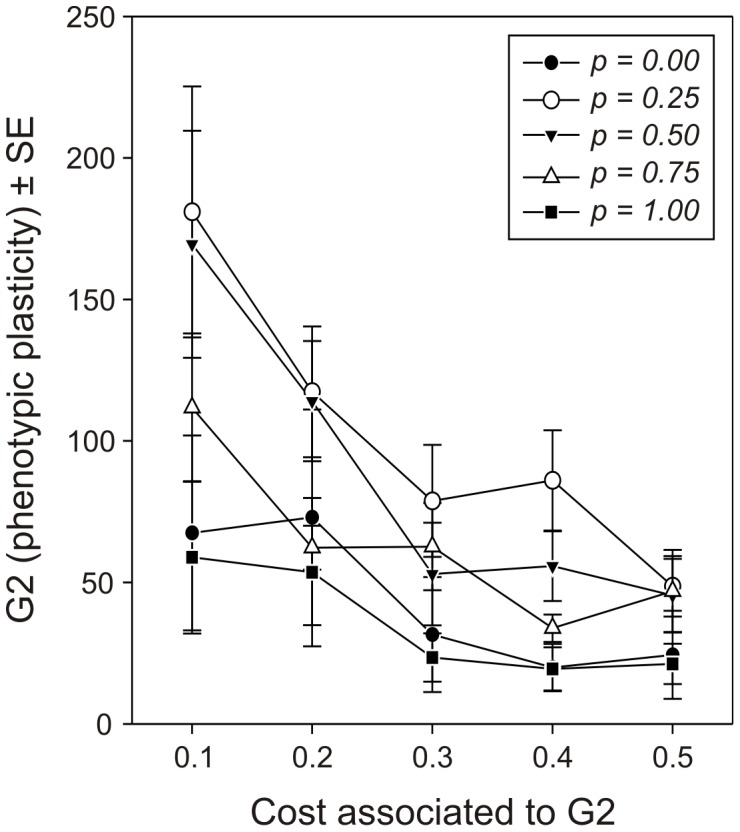
Effect of the cost of phenotypic plasticity and the probability for a parasitoid female to emerge on a host-containing habitat patch on the optimized values of the parameter G2. Average (±SE) optimized values of G2 defining the range of phenotypic plasticity (see Figure 1) for different values of the corresponding cost and different probabilities for the parasitoid females to start their life on a habitat patch.

**Figure 5:**
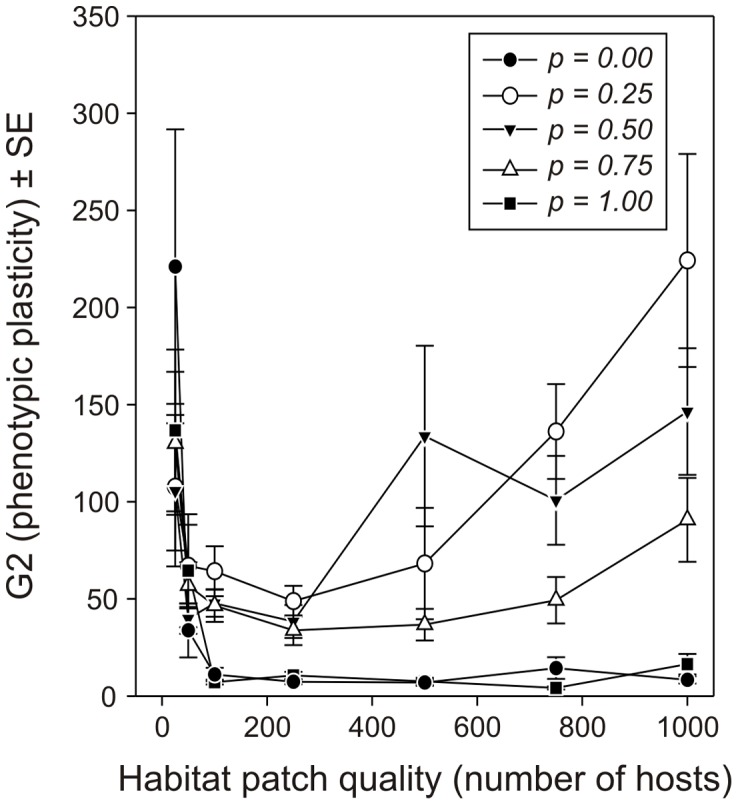
Effect of habitat patch quality and the probability for a parasitoid female to emerge on a host-containing habitat patch on the optimized values of the parameter G2. Average (±SE) optimized values of G2 defining the range of phenotypic plasticity (see Figure 1) for parasitoid females foraging for hosts in environments with different habitat patch qualities and having different probabilities to start their life on a habitat patch.

**Figure 6:**
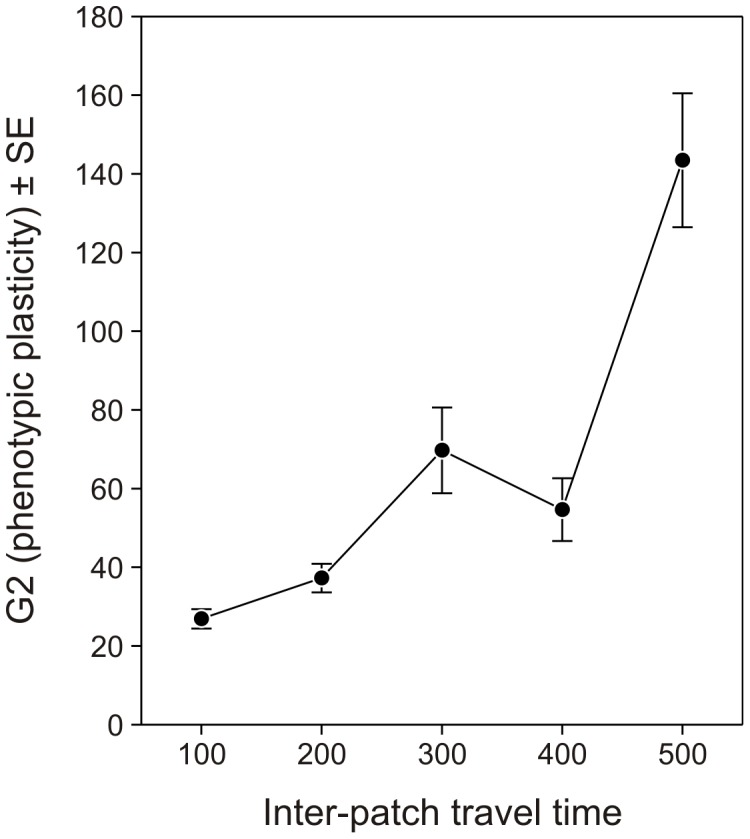
Effect of inter-patch travel time on the optimized values of the parameter G2. Average (±SE) optimized values of G2 defining the range of phenotypic plasticity (see Figure 1) for parasitoid females spending different times to travel between habitat patches.

The cost associated to phenotypic plasticity (G2) has an obvious effect (Fig. 4). The higher the cost, the less females should invest in being phenotypically plastic (F_4,736_ = 18.71, *p*<0.0001). However, when the cost is not too high, it becomes profitable to maintain some phenotypic plasticity, especially when females cannot be certain they will find hosts in their natal habitat patch (effect of the probability to be born on a host-containing habitat patch: F_4,736_ = 13.18, *p*<0.0001). The optimized level of phenotypic plasticity was also influenced by the quality of the habitat patches available in the environment (F_6,736_ = 18.02, *p*<0.0001; Fig. 5). That is, in low-quality habitat patches, it appeared that the probability that a female starts her life on a host-containing habitat patch was not important. In this case, females should have an intermediate level of phenotypic plasticity. The most likely reason for this is that, under these conditions, females spend a little time on small patches and a large amount of time travelling between habitat patches. Thus, whether or not they start their life by dispersing would not have a significant importance. With better-quality patches, however, being phenotypically plastic becomes important for females that are uncertain about starting their life on a host-containing habitat patch or not, *i.e.*, for intermediate values of *p*. Furthermore, the optimal level of phenotypic plasticity significantly increased with the time females spend travelling between patches (F_4,736_ = 34.48, *p*<0.0001; Fig. 6). When the time needed to reach each habitat patch is short, females spend most of their time on habitat patches containing hosts and are thus optimally allocating their resources to fecundity rather than to survival (see Fig. 2), without a need to maintain a substantial level of phenotypic plasticity. In contrast, when the time needed to reach each habitat patch increases, females should invest more into longevity (see Fig. 2), but should be able to trade this back for eggs when hosts are encountered. In these cases, phenotypic plasticity should be maintained.

**Figure 7:**
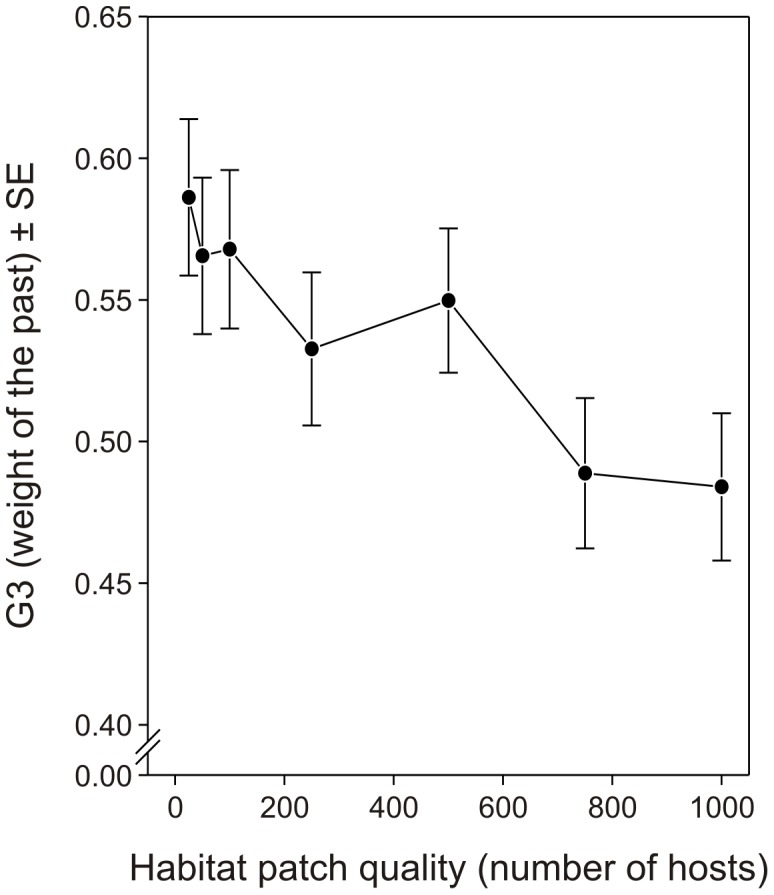
Effect of habitat patch quality on the optimized values of the parameter G3. Average (±SE) optimized values of G3 defining the weight of the past in the learning ability (see Figure 1) of parasitoid females foraging for hosts in environments with different habitat patch qualities.

Finally, in their learning process females that encounter habitat patches of better quality should forget their past more rapidly (F_6,736_ = 2.22, *p* = 0.0395; Fig. 7). Behaving in accordance with the marginal value theorem, females spend more time on better patches and, in this case, their past reproductive trajectory provides them with experience that progressively becomes less valuable.

## Discussion

Landscape structure is known to influence the reproductive success of insect parasitoids [10] and especially habitat fragmentation can have dramatic consequences, for example on biodiversity [72]. Such ecological and evolutionary consequences are still not fully studied and there is a serious need to understand how evolutionary processes can be affected by modifications and fragmentation of space [3], [73]. This is the reason why we developed a theoretical, simulation model to predict the optimal evolutionary responses of insect parasitoids foraging for hosts in environments with different levels of fragmentation. Our goal was thus to understand the evolutionary consequences of habitat fragmentation on the optimal reproductive strategy adopted by parasitic wasps foraging for hosts. Results indicate that, with increasing habitat fragmentation, wasp females will invest less into egg production and more into lifespan. Similar predictions are generated when females forage in habitats that show some level of stochasticity, at least in order to enable them to reach places where potential resources are. Such habitat stochasticity will also lead wasp females to maintain a certain level of phenotypic plasticity, especially if it does not entail an important cost for the animals and if the environment is highly fragmented with long travel times between patches of resources. Finally, foraging animals will likely be selected to learn the features of their habitat in order to maximise their reproductive success, especially if they are foraging in fragmented habitats consisting of small resource patches. Hence, habitat fragmentation seems to have potentially strong effects on the reproductive strategy adopted by foraging parasitic wasps, likely leading to evolutionary changes in several life-history traits that are linked to the spatial structure of their hosts (*e.g.*, dispersal, movements, patch time allocation, travel time between patches, resource allocation, etc.; [3]).

### The Life-history Trade-off

Results obtained are the consequence of a negative trade-off between longevity and reproduction, implying a competitive allocation of resources to either somatic maintenance or egg production. Such a trade-off is known to play an important role in decision making in many species [27], [74]. Here we accurately quantify how such a trade-off can shape the reproductive decision of animals foraging in fragmented habitats.

Although this has not been explicitly considered in our model, the evolutionary response to environmental selection implicitly assumes that there is significant genetic variation within wasp populations in the factors involved in the trade-off between survival and reproduction. Such variation has been experimentally observed by [12] between strains of *Asobara tabida*, a parasitoid of *Drosophila* larvae. Thanks to such genetic variation and to their ability to reciprocally trade instantaneously longevity for new eggs, our model predicts that wasp females will evolve ideally to an optimal reproductive decision, leading them to die at the exact moment in which they do not have any more egg to lay. However, this is neither what happens in real situations [25]–[26], nor what it is predicted by some other theoretical studies. Indeed, several models demonstrate that the majority of parasitoids should invest into higher egg load than the average number of hosts they encounter, and can thus end their life with a surplus of eggs. This is especially true when there is some stochasticity in the environment, leading to unpredictability in reproductive opportunity [13], [22], [25]–[26], [28], [50]. Environmental stochasticity is thus expected to influence the way resources should be allocated to either survival or reproduction [22], [50], which is what our model also predicts. More specifically, as has been suggested by other studies, our model indicates that environmental stochasticity should lead to maintenance of phenotypic plasticity in foraging animals, especially if the associated cost is not too high [55]. Even when females have to pay a significant cost, however, there remains a substantial optimal level of phenotypic plasticity that should be maintained, especially when animals have to forage in an unpredictable environment (see Fig. 4). This likely demonstrates that maintaining phenotypic plasticity is most likely an important component of the response of animals to the selective pressure coming from their fluctuant environment.

### Information Processing

Our model also predicts that the ability to use past experiences for dynamically modifying current reproductive strategies through a learning process is likely a useful feature for parasitic wasps that have to forage for hosts in fragmented habitats. In such cases, animals should ideally put more weight on the foraging information collected during their past reproductive trajectory in order to optimize their dynamic adaptation to the different characteristics of their habitat. Such a learning ability is most likely leading animals to avoid experiencing a risk of becoming either time- or egg-limited during their lifetime [17].

### Egg Maturation and Limitation

As was already mentioned above, our model makes several simplifications compared to real animals. Hence, several improvements could be considered in order to make the simulations more realistic. For example, since real animals cannot mature eggs instantaneously, eggs usually cannot be produced and laid immediately after finding a host ([24]; but see [62]). Such a delay in egg production is sometimes modelled by having newly matured eggs only laid in the next patch of resources visited [22]. Depending on host availability, a time delay in egg maturation is the reason why animals can experience either time- or egg-limitation in real situations, at least transiently [24]. Adding a delay (or an additional cost) in the model to account for egg maturation will increase the frequency of transient egg-limited phases in the life of simulated females, especially when their oviposition rate temporarily outstrips their egg maturation rate. In turn, this will lead females to globally invest more in higher egg load with lower longevity. However, as long as there remains a trade-off between longevity and reproduction, adding such a delay in egg maturation will not qualitatively change the main results presented in this work and the arguments developed here will remain essentially the same. Another simplification in our approach is that our modelled animals do not host-feed or consume sugar-rich foods, and hence do not gain additional resources to be used for survival or egg maturation [18], [74]. Adding a host-feeding behaviour to our model would most likely lead to a change in scale with a switch of the trade-off to the upper-right corner of the phase plane shown Fig. 1. Such additive energy can be allocated likewise to reproduction and survival, so this will most certainly not affect the qualitative predictions obtained and the main conclusions will remain the same [22], [31].

### Competition

Competition between females exploiting hosts can possibly influence optimal allocation strategies in parasitic wasps, but has also not been included in our model. Actually, competition between foragers could easily be added and finding optimal reproductive strategies with a genetic algorithm can be done in this case with the use of tournaments (*e.g.*, [65]). Competition could be added mainly in two different ways [22]. Firstly, it can occur within the hosts where larvae compete for host resources (*i.e.*, through superparasitism). If hosts are searched randomly, as this is the case in our model, adding this type of competition would simply result in a reduction in the expected payoff per egg, thus without significant qualitative changes in the main predictions of our model. Secondly, competition can occur between adults, leading females to completely or partially reject already attacked hosts. This would result in parasitoids experiencing globally poorer environments, thus investing in lower egg loads with higher survival rates. Here again, the qualitative predictions of our model will most likely remain unaffected. Under more complex situations, competition can lead to modify patch-leaving decision in parasitic females [75]–[77] with complex consequences in terms of resource allocation that remain to be analyzed.

### Evolutionary Consequences

Finally, our model does not consider potential evolutionary consequences of habitat fragmentation on the host strategies and thus on possible feedback in population dynamics affecting both hosts and parasitoids density and distribution. Yet, it is more than likely that changes in landscape structure should lead to significant coevolutionary effects on interacting species [3], with consequences in terms of population dynamics [78]–[79]. Thus, additional developments of the model are now being performed to allow host distribution strategies also to evolve. The expected results will provide new insight into the way habitat fragmentation can affect the evolution of interacting species.
